# Differential diagnosis between dementia and psychiatric disorders: Diagnostic criteria and supplementary exams. Recommendations of the Scientific Department of Cognitive Neurology and Aging of the Brazilian Academy of Neurology

**DOI:** 10.1590/S1980-57642011DN05040006

**Published:** 2011

**Authors:** Cássio M.C. Bottino, Analuiza Camozzato de Pádua, Jerusa Smid, Renata Areza-Fegyveres, Tânia Novaretti, Valeria S. Bahia, Working Group on Alzheimer’s Disease and Vascular dementia of the Brazilian Academy of Neurology

**Affiliations:** 1 Old Age Research Group, Institute of Psychiatry of Clínicas Hospital of the University of São Paulo School of Medicine (FMUSP), São Paulo SP, Brazil;; 2 Universidade Federal de Ciências da Saúde de Porto Alegre (UFCSPA), Hospital de Clínicas de Porto Alegre (UFRGS), Porto Alegre RS, Brazil;; 3 Cognitive and Behavioral Neurology Group of Clínicas Hospital of the University of São Paulo School of Medicine (FMUSP), São Paulo SP, Brazil;; 4 Cognitive and Behavioral Neurology Group of Clínicas Hospital of the University of São Paulo School of Medicine (FMUSP), São Paulo SP, Brazil;; 5 Faculdade de Filosofia e Ciências, Campus de Marília, da Universidade Estadual Paulista (UNESP), Marília SP, Brazil;; 6 Cognitive and Behavioral Neurology Group of Clínicas Hospital of the University of São Paulo School of Medicine (FMUSP), São Paulo SP, Brazil.

**Keywords:** dementia, Alzheimer's disease, depression, alcohol, psychoactive drug, guidelines, consensus, Brazil

## Abstract

In 2005, the Scientific Department of Cognitive Neurology and Aging of the Brazilian Academy of Neurology published recommendations for the diagnosis of Alzheimer's disease These recommendations were updated following a review of evidence retrieved from national and international studies held on PUBMED, SCIELO and LILACS medical databases. The main aims of this review article are as follows:

1) to present the evidence found on Brazilian (LILACS, SCIELO) and International (MEDLINE) databases from articles published up to May 2011, on the differential diagnosis of these psychiatric disorders and dementia, with special focus on Dementia due to Alzheimer's and vascular dementia, including a review of supplementary exams which may facilitate the diagnostic process; and
2) to propose recommendations for use by clinicians and researchers involved in diagnosing patients with dementia.

Differential diagnosis between dementia and other neuropsychiatric disorders should always include assessments for depression, *delirium*, and use of psychoactive substances, as well as investigate the use of benzodiazepines, anti-epileptics and pattern of alcohol consumption.

## Abstract (pt)

Em 2005, o Departamento Científico de Neurologia Cognitiva e do Envelhecimento da ABN publicou as recomendações para o diagnóstico da Doença de Alzheimer. Essas recomendações foram revisadas através de buscas em bases de dados PUBMED, SCIELO e LILACS, buscando evidências nacionais e internacionais sobre esses temas. Este artigo de revisão tem como objetivos:

1) apresentar as evidências encontradas em bases de dados brasileiras (LILACS, SCIELO) e internacionais (MEDLINE), até maio de 2011, sobre o diagnóstico diferencial desses transtornos psiquiátricos com demência, tendo como foco especial a demência de Alzheimer e a demência vascular, incluindo os exames complementares que podem auxiliar neste processo diagnóstico; e
2) propor recomendações que podem ser úteis a clínicos e pesquisadores envolvidos com o diagnóstico de pacientes com demência.

O diagnóstico diferencial entre demência e outros transtornos neuropsiquiátricos deve sempre incluir a avaliação de depressão, *delirium*, e o uso de substâncias psicoativas, tais como benzodiazepínicos, antiepilépticos e o padrão de consumo de bebidas alcoólicas.

## Introduction

In 2005, the Scientific Department of Cognitive Neurology and Aging of the Brazilian Academy of Neurology published recommendations for the diagnosis of Alzheimer's Disease.^1^ These recommendations were updated through consensus by Brazilian dementia experts. A review of the evidence was performed by searching for relevant articles on the PUBMED, SCIELO and LILACS medical databases using key words listed below in order to retrieve evidence on the themes available from national and international research.

According to diagnostic criteria for dementia (DSM-IV,^2^ CID-10^3^), other psychiatric disorders must be ruled out as the primary cause of cognitive or functional impairment prior to determining a diagnosis of dementia syndrome, a process which also applies to the diagnosis of the etiology of Alzheimer's and Vascular Diseases. The main differential diagnoses include: depression, *delirium*, use of psychoactive substances, including alcohol consumption.

The main aims of this review article are as follows:

[1] to present the evidence found on Brazilian (LILACS, SCIELO) and International (MEDLINE) databases from articles published up to May 2011, on the differential diagnosis of these psychiatric disorders with dementia, with special focus on Dementia due to Alzheimer's (AD) and Vascular Dementia (VD), including supplementary exams which may facilitate the diagnostic process; and
[2] to propose recommendations for use by clinicians and researchers involved in diagnosing patients with dementia.

### Depression

Depression is one of the main differential diagnoses for dementia. Therefore, the fact that dementia and depression can occur concomitantly should be considered, where depression as an antecedent of a dementia picture can represent a risk factor for, or prodrome to, dementia.^4^

Full anamnesis and assessment of psychic status are fundamental for reaching a differential diagnosis between depression and dementia. History of depressive episodes and prior treatment, the presence of medical and psychiatric comorbidities, use of medications and substances which can cause depressive symptoms, and the psychological characteristics of the patient at the time of the assessment, constitute the essential elements for the diagnostic rationale in most cases. The additional report by a family member of the subject's history of previous diseases and describing the characteristics and evolution of the individual's mental condition, are also important elements for screening depression in patients with cognitive deficits^5,6^ and for the differential diagnosis of dementia and depression. Table 1 below lists some clinical characteristics drawn from a review of the literature,^7^ which can assist clinicians and researchers in establishing their diagnostic rationale for differentiating AD from depression.

**Table 1:** Differential diagnosis between Alzheimer type dementia and depression.

Characteristics	Major Depressive Episode (MDE)	Alzheimer type Dementia (AD)
Diagnosis	Frequently meets criteria for MDE	Symptoms typically less intense than in MDE
Age at onset	Under or over the age of 60 years	Uncommon at less than 60 years of age
Onset	Typically acute	Insidious
Course	Fluctuations, often with congruent mood	Progressive
Memory complaints	Usually present	Variable
Mood	Depressive	Depressive or euthymic
Sleep-wake cycle	Often changed	Variable
Aphasia/apraxia/agnosia	Uncommon	Manifests as disease progresses
Memory	• Performance better than self-assessment • Performance better with cues for evoking • Intrusion of previously learned information atypical	• Performance worse than self-assessment • No performance improvement with use of cues • Intrusion of previously learned information upon evoking new material
Executive dysfunction	Typical	Variable, occurs later
Processing speed	Slowed	Normal
Effort	Reduces with cognitive demand, disproportionate compromise on more demanding tasks, "don’t know" responses	Usually normal

With regard to the differential diagnosis of vascular dementia and of depression, the overlap of the two conditions must be taken into account, particularly concerning the so-called "vascular depression".^7^ These two conditions often co-exist and share many common features, including cerebrovascular cerebral changes.^6,7^ Clinical presentations are also alike, with a broad spectrum of cognitive and functional changes which may occur in vascular depression that are also central in dementia pictures, such as executive dysfunction, attentional deficit, and slowing of information processing. Another key characteristic is the presence of apathy, as opposed to sadness, which is more common in vascular depression and also constitutes one of the more frequent neuropsychiatric symptoms in dementia. Loss of critical thought in patients represents a further obstacle in reaching a differential diagnosis of depression and vascular dementia. Moreover, the clinical picture of vascular depression as outlined above, may resemble frontal lobe syndrome, resulting in rupture of cortico-striatal-pallido-thalamo-cortical tracts, caused by cerebrovascular injury in these brain regions.^6-8^

According to the authors who proposed the concept of vascular depression, such patients evolve presenting with: more chronic symptoms (remission rates = 28 to 44%), worse response to treatment (response rates from 35% to 72%), greater recurrence of symptoms, greater functional incapacity, greater symptom severity, worse prognosis, greater risk of developing dementia.^8,9^

However, many questions remain controversial, namely: Is vascular depression a subtype of major depression? Are there specific symptoms? Are the clinical criteria proposed able to differentiate vascular depression from non-vascular depression? Although intriguing issues, the broader investigation into these questions goes beyond the scope of the present review, whose objective was to provide clinicians and researchers with consistent evidence for diagnosing dementia and depression.

The first option for assessing individuals with depression is screening instruments because they are both practical and quick to apply. Depression screening can be carried out using the "Geriatric Depression Scale" (GDS),^10,11^ or the "Centers for Epidemiologic Studies Depression Scale" (CES-D).^12,13^ Several other scales can be used for quantifying depressive symptoms, such as the Hamilton Depression Scale,^14,15^ Beck Depression Inventory,^16,17^ the Montgomery-Asberg Depression Scale,^14,15^ and Cornell Scale for Depression in Dementia.^18,19^ Another clinically relevant symptom found in elderly with dementia and/or depression is apathy, which can be assessed using other instruments such as the Neuropsychiatric Inventory (NPI),^20,21^ and the Apathy Evaluation Scale.^22,23^

A search of the Pubmed and LILACS databases using the uni-terms "GDS", "Brazil", "elderly", "EDG", and "Escala de Depressão Geriátrica", yielded 5 studies, whereas employing the terms "CES-D Scale", "Brazil" and "elderly" retrieved 4 studies. Finally, the uni-terms "Cornell Scale for Depression in Dementia" and "Brazil" returned six studies, "Neuropsychiatric Inventory", "NPI" and "Brazil" resulted in 7 studies, while 3 studies were identified matching the uni-terms "Apathy Scale" and "Brazil" where the article on the Portuguese version of the Apathy Evaluation Scale was published in the Dementia & Neuropsychologia journal. The search for studies on depression in Brazilian elderly yielded no validation studies in the elderly for the Montgomery-Asberg Depression Rating Scale, the Hamilton Depression Scale, or Beck's Depression Inventory.

Almeida & Almeida^11^ assessed 64 elderly diagnosed with Major Depression according to CID-10 and DSM-IV. The 15,10,4 and 1-item versions of the GDS were all tested. The authors concluded that using the GDS-15 and cut-off scores of 4/5 or 6/7 gave sensitivity of 92.7% and 80.5%, and specificity of 65.2% and 78.3%, for diagnosing Major depression, respectively (Class II Evidence).

In 2005, Paradela et al.^24^ assessed 302 elderly out-patients using the GDS-15. In their sample, 5.3% of the patients presented with depression and 11.6% dysthymia, based on DSM-IV. Adopting a cut-off score of 5/6 yielded sensitivity of 81.1% and specificity of 71.1% (Class II Evidence).

The CES-D scale was applied to 903 elderly residents of Juiz de Fora, between 2002 and 2003.13 Results were compared using the Brazilian version of the CES-D applied to a sub-sample of 446 elderly. The scale showed satisfactory internal consistency (α=0.86), sensitivity (74.6%), and specificity (73.6%), for the cut-off point >11. However, in the cited study, the CES-D produced a relatively high frequency of false positives compared with the GDS (33.8% *vs.* 15%) (Class II Evidence).

In 2007, Carthery-Goulart et al.^19^ assessed 29 patients with probable mild and moderate AD according to the NINCDS-ADRDA criteria, using the Brazilian version of the Cornell Scale for Depression in Dementia (CSDD). This scale was devised specifically to assess depressive symptoms in patients with dementia, being based on information reported by the examiner and family members or caregiver. The Brazilian version of the CSDD proved easy to apply, offering good intra-examiner (Kappa=0.77; p<0.001) and inter-examiner (Kappa=0.76 and p<0.001) reliability (Class IV Evidence)

In addition to the above-mentioned instruments to screen for or quantify depressive symptoms, interviews are also available that can help confirm a depression diagnosis which, although no substitute for a well-trained physician, may be useful in research settings or in cases of diagnostic doubt. The main limitation of such instruments for diagnosing dementia and/or depression, include the time needed for their application, a factor hampering their systematic use in routine clinical practice, as well as the absence of a comprehensive cognitive evaluation.

The "Structured Clinical Interview for DSM" (SCID) enables the diagnosing of mental disorders, with specific modules for each group of diseases such as mood disorders, using criteria from the DSM-IV. A Brazilian version of the SCID published in 1996^25^ is available. Although no specific validation studies were identified for the elderly or for diagnosing dementia patients, the scale has been used for assessing elderly with depression in research protocols.^26^ The Mini International Neuropsychiatric Interview (MINI) interview is another, relatively brief (15 to 30 mins), structured diagnostic instrument used for identifying psychiatric disorders based on DSM-IV and CID-10 criteria.^27^ The MINI has been used in a number of epidemiologic studies on clinical psychopharmacology, having been translated^28^ and validated in Portuguese and applied by resident medical students on a family medicine program.^29^

Another structured interview option was designed specifically for diagnosing dementia in elderly is the Structured Interview for Diagnosis of Mental Disorders in the Elderly (CAMDEX),^30^ which contains separate sections for assessing the patient and the family, plus a cognitive section (CAMCOG) comprising a brief neuropsychological battery. This interview, which is able to diagnose mental disorders such as dementia and depression according to CID-10 and DSM-IV criteria, has been translated and adapted in Portuguese and used in a number of Brazilian research centers.^31^ A study of 104 subjects (88% over 50 years of age) with complaints of cognitive decline^32^ found the Brazilian version of the CAMDEX to be effective for discriminating demented from depressive patients (Class IV Evidence).

Besides diagnostic instruments for diagnosing and screening, neuropsychological assessments can be used to differentiate dementia and depression, although no clear-cut neuropsychological pattern has been defined for the two conditions. Studies in this area have shown that some cognitive domains are more commonly affected in depression than in dementia, but these results were derived from comparisons of central tendencies in the samples studied. Nevertheless, no studies are available showing a consistent psychometric profile which can be recommended to reach a differential diagnosis. Changes in attention, executive functions and slowed information processing are the most frequently described cognitive alterations in studies involving depressed patients, particularly those with late onset depression - occurring after 60 or 65 years of age (Class IV Evidence).^33-36^

Currently, there is insufficient evidence to recommend structural or functional neuroimaging exams for differential diagnosis between dementia and depression (Class IV Evidence).^37-39^

Recommendation: Versions of the GDS containing 15 items can be considered for screening depression in elderly in Brazil (B Level Evidence). The CES-D Scale can be considered another option for screening (Class C Evidence). The Cornell scale can be used in order to quantify the depressive symptoms in patients with dementia (Class U Evidence). The CAMDEX interview can be employed for reaching a differential diagnosis between depression and dementia (Level U Evidence). The use of neuropsychological tests can assist clinical differentiation between dementia and depression (Level U Evidence). Current evidence suggests that neuroimaging exams are not recommended (Level U Evidence).

### Delirium

*Delirium*, or an acute confusional picture, is frequent in patients aged older than 65 years, and is associated to increased mortality and morbidity.^40,41^ *Delirium* is typically characterized by acute onset (hours or days) of change in conscience and cognitive and attentional decline which is oscillatory in nature, with alterations in perception (illusions, hallucinations), triggered by cerebral or systemic disease. The two forms of *delirium* are hypoactive and hyperactive. Hypoactive delirium is frequent in older adults and a form that often goes underdiagnosed.^40^

Several scales have been proposed to aid diagnosis of *delirium* conditions, especially to help screen for the condition in hospitalized patients. The main scales in use are: *Confusion Assessment Method* (CAM), *Delirium Rating Scale, Memorial Delirium Assessment Scale* and *NEECHAM Confusion Scale.*^42^ However, the Confusion Assessment Method (CAM) is the only scale validated for use in Brazil.^43^

In 2001, Fabri et al.^43^ applied the CAM to 100 elderly subjects in an Emergency Room setting for the objective assessment of *delirium*, diagnosed according to DSM-IV. They found sensitivity of 94.1% and specificity of 96.3% whereas inter-examiner reliability (Kappa) in a sub-sample of 24 patients was 0.70 (Class II Evidence).

**Recommendations** - The CAM can be recommended to help diagnose *delirium* in Brazilian elderly patients (Level C Evidence).

### Other mental disorders possibly associated with dementia

Use of benzodiazepines, anti-convulsants, and alcohol abuse/dependence must be investigated in the assessment of patients with dementia.^44^

Review and meta-analyses have shown that chronic benzodiazepine use can lead to cognitive impairments which persist for months following discontinuation of their use (Class II Evidence).^45,46^

Elderly individuals are most susceptible to the effects of anti-epileptic medications because of pharmacokinetic factors. Among this class of drugs, the medication causing the most severe cognitive and behavioral dysfunctions is Topiramate.^47,48^ In a double-blind, placebo-controlled trial in 188 cognitively-healthy individuals with a mean age of between 40 and 47 years, Loring et al.^49^ showed a dose-related decline in cognitive function with Topiramate use (Class II Evidence).

In the Brazilian Scientific Literature, two case studies show the emergence of neuropsychiatric symptoms triggered by Topiramate use.^50,51^ However, no well-structured studies, albeit Brazilian or International, on the cognitive effects of Topiramate use in elderly individuals are available.

Chronic consumption of large amounts of alcohol produces a glutamate-induced cytotoxic effect, leading to permanent neuronal injury which predisposes such individuals to neuropsychiatric disorders, including dementia. Moreover, chronic alcoholics often have nutritional deficits which lead to the same problems.^52^

In the ensuing section, the diseases associated to cognitive compromise related to chronic alcohol use are described:

#### ALCOHOL-RELATED DEMENTIA

Progressive cognitive decline can occur in chronic alcoholics as a result of alcohol dependence, irrespective of nutritional deficits. The toxic effect of alcohol predominantly affects the frontal superior association cortex, the hypothalamus and the cerebellum. In addition, structural changes in myelin can take place although these may be reversible following abstinence.^53^

The clinical criteria for alcohol-related dementia according to Oslin et al.^54^ include:

- Dementia diagnosis performed at least 60 days after last exposure to alcohol:
- Minimum 35 standard doses for men and 28 for women per week for over 5 years and;
- Significant alcohol abuse within 3 years of onset of cognitive decline.

#### WERNICKE-KORSAKOFF SYNDROME

The most frequent nutritional deficiency resulting from chronic alcohol use is vitamin B1 deficiency (Thiamin) which can induce Wernicke-Korsakoff syndrome.

Wernicke's syndrome is characterized by the following symptoms (associated or isolated): mental confusion, abnormality in extrinsic ocular movement and gait ataxia. If untreated, the patient can evolve to death or to Korsakoff's Syndrome.^55^

Korsakoff's syndrome is clinically characterized by episodic memory deficit with the hallmark presence of confabulations, variable compromise in semantic memory, nystagmus and ataxic gait.^55,56^

Findings on structural neuroimaging include predominantly frontal cortical atrophy and reduced volume of the thalamus and mammillary bodies.^57^

Besides the known etiology of alcoholism, Korsakoff's Syndrome can also occur in patients with persistent vomiting, gastroplasty, puerperium, infection, intoxication or other chronic diseases. Genetic risk factors associated to thiamin deficiency have also been investigated.^58^

#### MARCHIAFAVA-BIGNAMI DISEASE

Marchiafava-Bignami disease is rare and generally diagnosed in alcoholics. It can manifest in acute, subacute or chronic forms. The symptoms range from dementia, muscular hypertonia, epileptic episodes and dysphagia, with patients often evolving to a comatose state. This disease has a high lethality rate.

Neuroimaging exams disclose prominent atrophy of the corpus collosum, with varying degree of necrosis and cystic formations.^59^

Bello & Schultz^60^ assessed the prevalence of reversible dementia cases including alcohol-related dementia among patients seen in specialized outpatient clinics. Of the 340 patients treated between 1999 and 2009, 19.17% had potentially reversible dementia and among this subgroup, 3% had dementia secondary to alcohol use. In a population-based study assessing the prevalence and causes of dementia in elderly residents of São Paulo, a 4.7% prevalence of dementia related to alcohol use was found in a sample of 107 patients diagnosed with dementia.^61^ In another analysis by the same population-based study, Hirata et al.^62^ found that 9.1% of these elderly had problems associated to alcohol use according to the CAGE^63^ screening scale. These elderly subjects exhibited worse cognitive and functional impairment, indicating a higher risk of dementia diagnosis.^62^ In another population-based study conducted in Ribeirão Preto, Lopes et al.^64^ found a similar association between alcohol abuse and cognitive and functional impairment, but suggested a protective effect of moderate alcohol use. The association of dementia with chronic and abusive alcohol use has been consistently reported in epidemiological studies (Class II Evidence), while data points to a possible protective effect of moderate alcohol consumption.^65^ These results highlight the importance of systematic screening for alcoholic beverage consumption among patients with suspected dementia.

No validation study was found for CAGE use in Brazil, but the scale has been used as an instrument for screening alcohol-related problems in elderly populations.^62,64^ Other instruments for screening alcohol-related problems can be used such as the Alcohol Use Disorder Identification Test (AUDIT),^66^ translated and validated for use in Brazil,^67^ and recently used in a population of elderly men.^68^ Another screening scale option is the Michigan Alcoholism Screening Test" (MAST)^69^ which has been validated for use in the elderly^70^ and also in the Brazilian elderly male population.^71^

Therefore, CAGE, AUDIT and MAST scales (Class II Evidence) can be employed for screening problems associated with alcohol use, having been validated and/or assessed in representative samples of elderly in Brazil.

**Recommendations** - Chronic use of benzodiazepines (Evidence Level B) and anti-epileptic drugs (Evidence Level U), especially Topiramate should be investigated in elderly with cognitive impairment. Abusive alcohol use and dependency can cause dementia, and the CAGE, AUDIT and MAST scales can be used for the screening of alcohol-related problems in the elderly (Evidence Level B).

## Conclusion

Differential diagnosis between dementia and other neuropsychiatric disorders should always include assessments for depression, *delirium*, and use of psychoactive substances, as well as investigate the use of benzodiazepines, anti-epileptics and pattern of alcohol consumption. Current diagnostic criteria for dementia require the exclusion of other neuropsychiatric disorders, yet no supplementary exams to reliably provide this differential diagnosis are available. However, rigorous clinical assessment coupled with the use of screening instruments validated for use in Brazil can improve clinicians and researchers' effectiveness in reaching a differential diagnosis of dementia and other psychiatric disorders.

## Figures and Tables

**Chart 1:** Diagnostic criteria for vascular depression.

Presence of two cardinal characteristics:
• Evidence of risk factor or vascular dementia.
• Onset of depression later than 65 years of age or change in course of depression after vascular disease in individuals with early onset of depression.
Presence of some secondary characteristics:
• Cognitive compromise, psychomotor slowing, poor depressive ideation, limited insight, no family history of mood disorders, and disability.

## GROUP RECOMMENDATIONS IN ALZHEIMER’S DISEASE AND VASCULAR DEMENTIA OF THE BRAZILIAN ACADEMY OF NEUROLOGY

**Amauri B. da Silva** [UNINEURO, Recife (PE)]; **Ana Cláudia Ferraz** [Serviço de Neurologia do Hospital Santa Marcelina (SP)]; **Antonio Lúcio Teixeira** [Departamento de Clínica Médica, Faculdade de Medicina da Universidade Federal de Minas Gerais, Belo Horizonte (MG)]; **Ayrton Roberto Massaro** [Instituto de Reabilitação Lucy Montoro (SP)]; **Benito Pereira Damasceno** [Departamento de Neurologia da Universidade Estadual de Campinas (SP)]; **Carla Tocquer** [Universidade Federal do Rio de Janeiro (RJ)]; **Carlos Alberto Buchpiguel** [Departamento de Radiologia, Faculdade de Medicina da Universidade de São Paulo (SP)]; **Charles André** [Faculdade de Medicina - UFRJ; SINAPSE Reabilitação e Neurofisiologia (RJ)]; **Cláudia C. Godinho** [Serviço de Neurologia do Hospital de Clínicas de Porto Alegre, Universidade Federal do Rio Grande do Sul (RS)]; **Cláudia Sellitto Porto** [Grupo de Neurologia Cognitiva e do Comportamento da Faculdade de Medicina da USP (SP)]; **Delson José da Silva** [Núcleo de Neurociências do Hospital das Clínicas da Universidade Federal de Goiás (UFG); Instituto Integrado de Neurociências (IINEURO), Goiânia (GO)]; **Denise Madeira Moreira** [Departamento de Radiologia Faculdade de Medicina - UFRJ; Setor de Radiologia - IN DC - UFRJ (RJ)]; **Eliasz Engelhardt** [Setor de Neurologia Cognitiva e do Comportamento - IN DC - CDA/IPUB - UFRJ (RJ)]; **Elza Dias-Tosta** [Presidente da Academia Brasileira de Neurologia, Hospital de Base do Distrito Federal (DF)]; **Emílio Herrera Junior** [Departamento de Medicina Interna, Faculdade de Medicina de Catanduva (SP)]; **Francisco de Assis Carvalho do Vale** [Universidade Federal de São Carlos (UFSCar), Departamento de Medicina (DMed) (SP)]; **Gabriel R. de Freitas** [Instituto D'or de Pesquisa e Ensino; Universidade Federal Fluminense (RJ)]; **Hae Won Lee** [Instituto de Radiologia, Hospital das Clínicas da Faculdade de Medicina da Universidade de São Paulo e Hospital Sírio-Libanês (SP)]; **Ivan Hideyo Okamoto** [Departamento de Neurologia e Neurocirurgia; Instituto da Memória - Universidade Federal de São Paulo - UNIFESP (SP)]; **João Carlos Barbosa Machado** [Aurus IEPE - Instituto de Ensino e Pesquisa do Envelhecimento de Belo Horizonte; Faculdade de Ciências Médicas de Minas Gerais (FCMMG), Serviço de Medicina Geriátrica do Hospital Mater Dei (MG)]; **José Antonio Livramento** [Laboratório de Investigação Médica (LIM) 15, Faculdade de Medicina da Universidade de São Paulo (SP)]; **José Luiz de Sá Cavalcanti** [Departamento de Neurologia - INDC - UFRJ; Setor de Neurologia Cognitiva e do Comportamento - INDC - UFRJ (RJ)]; **Letícia Lessa Mansur** [Grupo de Neurologia Cognitiva e do Comportamento do Departamento de Neurologia da FMUSP; Departamento de Fisioterapia, Fonoaudiologia e Terapia Ocupacional da Faculdade de Medicina da USP (SP)]; **Márcia Lorena Fagundes Chaves** [Serviço de Neurologia do Hospital de Clínicas de Porto Alegre, Universidade Federal do Rio Grande do Sul (RS)]; **Márcia Radanovic** [Laboratório de Neurociências - LIM27, Departamento e Instituto de Psiquiatria da Faculdade de Medicina da Universidade de São Paulo (FMUSP) (SP)]; **Márcio Luiz Figueredo Balthazar** [Universidade Estadual de Campinas (UNICAMP), Faculdade de Ciências Médicas (FCM), Departamento de Neurologia (SP)]; **Maria Teresa Carthery-Goulart** [Grupo de Neurologia Cognitiva e do Comportamento do Departamento de Neurologia da Faculdade de Medicina da USP; Centro de Matemática, Computação e Cognição, Universidade Federal do ABC (SP)]; **Mônica S. Yassuda** [Grupo de Neurologia Cognitiva e do Comportamento do Departamento de Neurologia da Faculdade de Medicina da USP; Departamento de Gerontologia, Escola de Artes, Ciências e Humanidades da USP (EACH/USP Leste) (SP)]; **Nasser Allam** [Universidade de Brasília (UnB), Laboratório de Neurociências e Comportamento, Brasília (DF)]; **Norberto Anizio Ferreira Frota** [Universidade de Fortaleza (UNIFOR), Serviço de Neurologia do Hospital Geral de Fortaleza (HGF) (CE)]; **Orestes Forlenza** [Laboratório de Neurociências - LIM27, Departamento e Instituto de Psiquiatria da Faculdade de Medicina da Universidade de São Paulo (FMUSP) (SP)]; **Paulo Caramelli** [Departamento de Clínica Médica, Faculdade de Medicina da Universidade Federal de Minas Gerais, Belo Horizonte (MG)]; **Paulo Henrique Ferreira Bertolucci ** [Universidade Federal de São Paulo (UNIFESP), Setor de Neurologia do Comportamento - Escola Paulista de Medicina, São Paulo (SP)]; **Regina Miksian Magaldi** [Serviço de Geriatria do Hospital das Clínicas da FMUSP, Centro de Referencia em Distúrbios Cognitivos (CEREDIC) da FMUSP (SP)]; **Renato Anghinah** [Grupo de Neurologia Cognitiva e do Comportamento do Hospital das Clínicas da Faculdade de Medicina da Universidade de São Paulo (FMUSP); Centro de Referência em Distúrbios Cognitivos (CEREDIC) da FMUSP (SP)]; **Ricardo Nitrini** [Grupo de Neurologia Cognitiva e do Comportamento do Hospital das Clínicas da Faculdade de Medicina da Universidade de São Paulo (FMUSP); Centro de Referência em Distúrbios Cognitivos (CEREDIC) da FMUSP (SP)]; **Rodrigo Rizek Schultz** [Setor de Neurologia do Comportamento do Departamento de Neurologia e Neurocirurgia da Universidade Federal de São Paulo, Núcleo de Envelhecimento Cerebral (NUDEC) - Instituto da Memória (UNIFESP) (SP)]; **Rogério Beato** [Grupo de Pesquisa em Neurologia Cognitiva e do Comportamento, Departamento de Medicina Interna, Faculdade de Medicina, UFMG (MG)]; **Sonia Maria Dozzi Brucki** [Grupo de Neurologia Cognitiva e do Comportamento da Faculdade de Medicina da Universidade de São Paulo; Centro de Referência em Distúrbios Cognitivos (CEREDIC) da FMUSP; Hospital Santa Marcelina (SP)]; **Ylmar Corrêa Neto** [Universidade Federal de Santa Catarina (UFSC), Departamento de Clínica Médica, Florianópolis (SC)].
